# Age at League Entry and Early All-Cause Mortality among National Football League Players

**DOI:** 10.3390/ijerph182413356

**Published:** 2021-12-18

**Authors:** Bhavneet Walia, Brittany L. Kmush, Justin Ehrlich, Madeline Mackowski, Shane Sanders

**Affiliations:** 1 Department of Public Health, Syracuse University, Syracuse, NY 13244, USA; bwalia@syr.edu (B.W.); blkmush@syr.edu (B.L.K.); mahilton@syr.edu (M.M.); 2 Department of Sport Management, Syracuse University, Syracuse, NY 13244, USA; jaehrlic@syr.edu

**Keywords:** NFL player health, sports, American football, mortality, league health and safety policy, cumulative head impact, Cox proportional hazard regression, risk factors, retired athletes

## Abstract

Background: A growing body of research suggests that American football players are exposed to higher cumulative head impact risk as competition level rises. Related literature finds that head impacts absorbed by youth, adolescent, and emerging adult players are associated with elevated risk of long-term health problems (e.g., neurodegenerative disease onset). Most National Football League (NFL) players enter the League as emerging adults (18–24 years old), a period of continued cognitive and overall physical development. However, no prior research has studied the effect of age-at-entry on long-term NFL player health. Hypothesis/Purpose: This study assesses whether early NFL player age-at-entry is associated with increased risk of early all-cause mortality, controlling for player position, BMI, year-of-entry, birth year, and NFL Draft round (expected ability upon League entry). Study Design: This retrospective cohort study included 9049 players who entered the NFL from 1970–2017 and subsequently played at least one game. The variables whether deceased, age-at-death, age-at-entry, and controls were collected from Pro Football Reference website, a leading data site for American football that has been used extensively in the literature. Data collection began on 13 July 2017, and follow-up ended on 1 July 2018. Statistical analysis was performed from 10 March 2020 to 3 August 2020. Data was validated by checking a large sub-sample of data points against alternative sources such as NFL.com and NFLsavant.com. Methods: Cox proportional hazards regression models were used to examine variation in death hazard by NFL player age-at-entry, conditional upon a full set of controls. Results: Conditional on controls, Cox regression results indicate that a one-year increase in age-at-entry was significantly associated with a 14% decreased hazard-of-death (H.R., 0.86; 95% CI, 0.74–0.98). Among relatively young entering players, the increased hazard appears to be concentrated in the first quartile of players by age at League entry (20.2 to 22.3 years). Players *not* in this quartile exhibited a decreased hazard-of-death (H.R., 0.74; 95% CI, 0.57–0.97) compared with players who entered at a relatively young (first quartile) age. Conclusion: An earlier age-at-entry is associated with an increased hazard-of-death among NFL players. Currently, the NFL regulates age-at-entry only indirectly by requiring players to be 3 years removed from high school before becoming NFL Draft-eligible. Implementing a minimum age at entry for NFL players of 22 years and 4 months at beginning of season is expected to result in reduced mortality. What is known about this subject? There are no prior studies on the effects of NFL player age-at-entry on early mortality risk. What this study adds to existing knowledge: This study determines whether entering the NFL at an age of physical and physiological development is related to early mortality risk.

## 1. Introduction

Early entry into youth tackle football is associated with increased risk for cognitive impairment in later life, including long-term cognitive, neuropsychiatric, and neurologic disturbances, even in individuals who only played high school football [1,2,3,4,5,6]. Age of first exposure to tackle football is associated with earlier neurobehavioral symptom onset among former players diagnosed with CTE [7]. However, limited contradictory evidence exists, albeit from an older cohort. A cohort study of males graduating from a Wisconsin high school in 1957 finds no evidence that having played high school football elevates the risk of later-life cognitive impairment [8]. Venkataramani et al. find no evidence of elevated early mortality risk for regular National Football League (NFL) players versus NFL replacement players [9]. As the study points out, however, each replacement player endured an extensive American football career.

There are reasons for concern about the long-term health risks faced by youth and emerging adult American football players, whose neurological and muscular systems remain in development. Baillargeon et al. find that adolescents face greater short- and long-term neurophysiological effects from concussion than adults [10] Silveri states, “Brain maturation and associated improvements in decision-making continue into the second decade of life, reaching plateaus within the period referred to as ‘emerging adulthood’ (18–24 years)” [11]. Silveri further states that an individual reaches “neurobiological adulthood” only during this plateau period [11]. Pellman et al. find that the rate of neuropsychological recovery following concussion was higher in older players [12].

The extant literature has found a strong link between brain injury and tauopathies, the accumulation, and misfolding of neurofibrillary tangles. In turn, tauopathies contribute to the onset of neurodegenerative disease such as Alzheimer’s Disease and chronic traumatic encephalopathy, which, in turn, present elevated mortality risks for an individual. Further, studies suggest that American football players experience substantially higher cumulative head impact levels per exposure as the competitive level of play increases, where cumulative head impact is typically defined as the cumulative force of head impacts of at least 10 g of peak linear acceleration [13,14]. American football is a competitive labor tournament in which bigger, faster, and stronger players are disproportionately selected into further rounds of competition. Daniel et al. find that average estimated head impact counts per season rise from 107 in youth football, to 565 in high school football, to 1000 in college football [14]. At those respective levels-of-play, the estimated median rotational acceleration resulting from head impact also rises from 672 radians/s, to 903 radians/s, and to 981 radians/s, respectively. Evidence suggests that this trend continues into the NFL level, in which there are an estimated 6.61 concussions per 1000 in-game athlete exposures [15], compared with 2.5 at the NCAA level [16]. Therefore, we expect a 21-year-old NFL player to face far greater head impact risk than his age and position peers playing NCAA football.

Younger NFL players face two potential elevated risk factors to contend with: the level of play imposes a greater cumulative head impact risk, and their younger age may afford them less protection against the long-term neuropsychological player health effect of such hits. Without an explicit minimum age policy, the NFL has allowed the drafting of many players who are in the first half of emerging adulthood (first half of the period from 18–24 years old) [11], a period in which the brain is typically not yet mature. Given the findings of Baillargeion et al. the compounding factors of age and environment-of-play may impose long-term player health consequences that relate to player developmental fitness and associated response to NFL-level cumulative head impact [10]. The short- and long-term health risks associated with NFL play may be greater for players who enter the League at a younger age. The League has no formal age restriction, only requiring that players be at least three years removed from high school. As such, some players may not enter the League without the full protective benefit of neuromuscular or cognitive maturity. Two extreme examples, in recent years, are Amobi Okoye and Tremaine Edmunds, who were both drafted at 19 years old and played in NFL games shortly after reaching 20 years old.

Player health risks in American football represent a longstanding issue in research and public discourse [17,18]. Prior research demonstrates that American football league policies are becoming more active in regulating player health via league health and safety policy changes [9,19]. Moreover, it has been shown that league policies can lead to tangible improvements in player health [20,21]. Herein, we examine whether age-at-entry is associated with earlier all-cause mortality among NFL players. We studied 9049 players who were drafted into the NFL between 1970 and 2017. This time period allows for a long panel of former player cohorts but not so long as to confound the study with stark changes in the nature of certain playing positions (e.g., the advent of the linebacker heavy three–four defensive set in the early 1970s; the diminished importance of the fullback position in recent decades). This represents the first study to systematically examine the association between age and mortality in emerging adults engaged in professional American football play.

The practical implications of the study are as follows: age-at-entry represents a choice variable for the NFL. For example, the NBA enforces an explicit age at league entry. This study finds significant evidence that an advanced age at league entry is protective of mortality in a study that features comprehensive controls. In a high impact sport such as American football, it is vital to continue examining the age-specific impacts of play and the age-specific impacts of level-of-play upon player health.

## 2. Methods

### 2.1. Data Collection

The present cohort study uses data from Pro Football Reference (PFR), an online, open-access database published by Sports Reference LLC. The site provides detailed player information (game statistics, biometric information, and biographical information) for more than 25,000 former and current NFL players whose careers span the universe of NFL seasons (1922-present). We retrieved all data from Sports Reference, a leading sports data site. Variables in the analysis include age at start of first NFL season, date of birth, date of death (if applicable), round selected in NFL player draft, primary NFL playing position group, NFL games played, and listed body mass index (BMI) measured as mass in kilograms per square of height in meters as measured during most recent active NFL season. These variables help us address the null and alternative hypotheses of the study, namely:

**Null** **Hypothesis:** *Age at entry into the NFL is not associated with all-cause mortality among former NFL players*.

**Alternative** **Hypothesis:** *Age at entry into the NFL is associated with all-cause mortality among former NFL players*.

**Figure 1:**
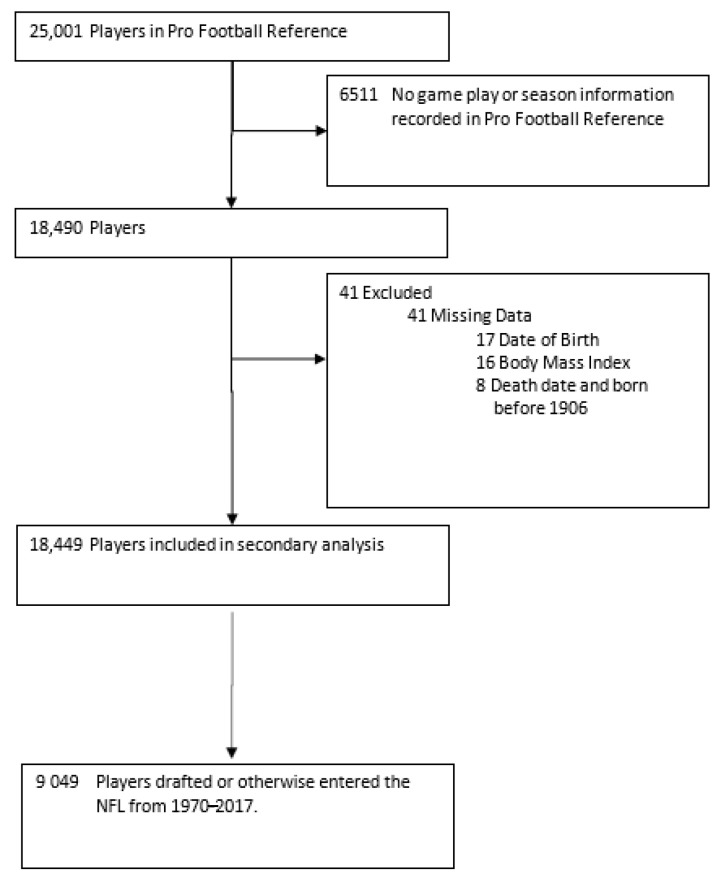
Cohort flow chart.

The risks associated with exposure to play were controlled via a combination of player-position cluster indicator variables and the number of career NFL games played for each observed player. This approach is similar to the novel Cumulative Head Impact Index approach to controlling for NFL game exposure early mortality risks [2,22,23]. Specifically, NFL games played controls for NFL game exposure, while position cluster indicator variables control for variation in expected per game risk by position of play (due, e.g., to variation in head impact risk per game played or to physiological attributes that vary by position and are not strongly associated with BMI). Initial data collection for the study began on 13 July 2017, and follow-up ended on 1 July 2018. Participants include all former and current NFL individuals who were fully observed in the Pro Football Reference database. The eligibility criterion was having played in at least one regular season NFL game. The sample size calculation is provided in Figure 1. This study overcomes sample selection bias in several past studies of American football player mortality by considering the whole population of NFL players. However, there may be biases related to why some players are not fully observed in the data set. As all data were collected from a public data source, the Syracuse University Institutional Review Board exempted the study from review, oversight, and informed consent. The study follows Strengthening the Reporting of Observational Studies in Epidemiology (STROBE) guidelines.

### 2.2. Calculation of Age-at-Entry into the NFL

**Figure 2:**
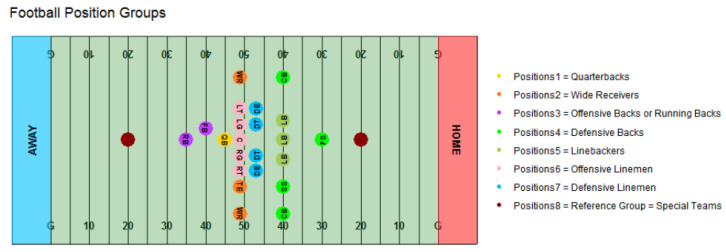
Football position groups. FS = free safety; CB = cornerback; SS = strong safety; LB = linebacker; DE = defensive end; DT = defensive tackle; RB = running back; FB = full back; QB = quarterback; WR = wide receiver; LT = left tackle; LG = left guard; C = center; RG = right guard; RT = right tackle; TE = tight end.

NFL age-at-entry was calculated as age (in years) on August 1 of the player’s first year of NFL game play. Age-at-entry was considered as both a continuous and categorical variable in alternation. For the categorical variable, all sampled players were sorted by age and placed into an age-at-entry quartile. For example, the first quartile constitutes players aged 20.2 to 22.3 years. A binary variable was then coded to reflect whether or not a player was in the first quartile of the continuous age-at-entry variable (i.e., 22.3 years or less upon entry). We use this quartile cut-off because it (a) is taken from the distribution rather than arbitrarily chosen by the researchers, (b) represents an early age-at-entry by definition, and (c) falls in the heart of the age range of emerging adulthood. The categorical age-at-entry variable considers the possibility that an advanced age-at-entry is protective only to a point (e.g., a point roughly coinciding with near cognitive maturity). As in Montenigro et al. [22] position clusters were assigned according to helmet accelerometer study groupings with the advantage that the present study is able to include special teams as a position cluster given our black-box, indicator variable approach to controlling for position cluster [21]. Position clusters are given in Figure 2.

Player-position group indicator variables were created using the generate function in Stata (Stata, Version 14; StataCorp LLC, College Station, TX, USA) [24], as was BMI. BMI was calculated based on listed playing height and mass from PFR according to the standard BMI formula (mass in kilograms divided by the square of height in meters). All other study variables, including age-at-death, were acquired and analyzed directly as reported in PFR.

### 2.3. Statistical Analysis

We compared demographic characteristics across position categories. A Chi-squared test was used for categorical variables, ANOVA was used for normally distributed continuous variables, and a Kruskal—test was used for non-normally distributed continuous variables. A set of Cox proportional hazard regression models was specified to calculate mortality hazard, conditional upon age-at-entry, player-position group, birth year (alternatively, decade cohort of birth), and round of selection in the player draft, a surrogate for skill level upon League entry. If a player entered the NFL by signing a contract as an undrafted free agent, he was assigned a draft selection round of one greater than the total number of Draft rounds that season.

Study variables were specified a priori based on previous work (see, e.g., Kmush et al., 2017 [23]). Players with missing data were eliminated from the analysis, as detailed in Figure 2. Sensitivity analyses were completed by specifying alternative control variables in stepwise Cox proportional hazards regression models. Statistical analysis was performed from 10 March 2020 to 3 August 2020. All tests were two-sided, where *p*-values ≤0.05 were considered statistically significant. All analyses were completed in Stata, Version 14 (StataCorp LLC, College Station, TX, USA) [24].

### 2.4. Ethical Approval

Data was collected from a publicly available website (not private information) and was determined by the Syracuse University Institutional Review Board to not require review and oversight.

## 3. Results

There were a total of 25,001 NFL players listed in the PFR, wherein 6511 were on a team roster but did not compete in a regular season NFL game, and 41 had missing data. Of the remaining 18,449 players who played in at least one NFL game between 1922 and 2017 and were fully observed in the data set, 9049 were drafted or otherwise acquired into the NFL between 1970 and 2017 and played in at least one regular season game (Figure 2). These 9049 individuals were included in the analysis.

**Figure 3:**
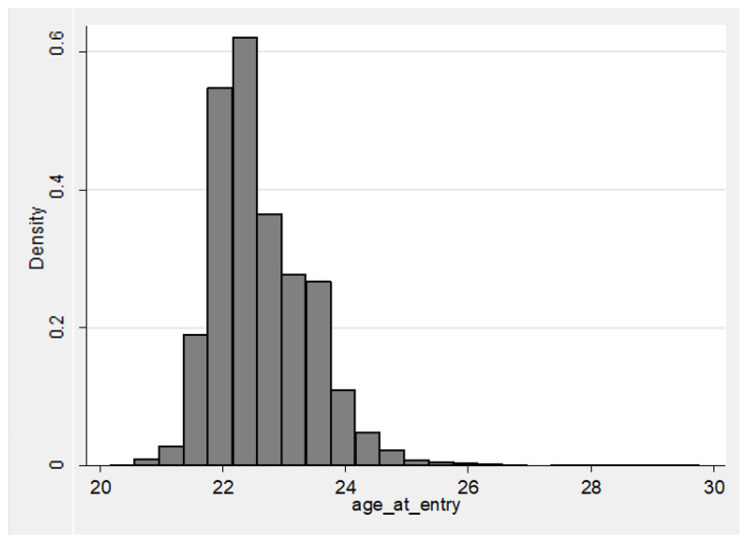
Age at NFL entry histogram (players entering from 1970–2017) (*n* = 9049).

As of 1 July 2018, the mean (SD) age of NFL players who entered the league from 1970–2017 was 46.6 (13.5) years, where 365 (4.0%) had died as of the date of data collection. Mean (SD) age at NFL entry was 22.9 (0.8) years, with an approximately normal distribution (Figure 3).

There is a fairly substantial range in NFL age-at-entry, from 20.2 years to 29.4 years. This range spans approximately 11.5 standard deviations, with greater length on the right tail, given the NFL’s indirect age rule (which soft-censors the left tail). As our study represents essentially a census of NFL players with game experience over the data period, we need not restrict outliers from the analysis, as in a sample-based inferential study. Figure 3, displayed previously, shows the distribution of NFL age-at-entry from 1970 to 2017. Each demographic variable was significantly different across playing position groups (*p* < 0.05, Table 1).

Table 2 displays the output of four Cox regression models. Output is displayed in hazard-ratio form.

Conditional upon birth year or birth decade, player position groups, NFL Draft selection round, BMI, career NFL games, and career NFL games squared, each of these four specifications reveals that an advanced age-at-entry was associated with a significant protective effect upon a player’s hazard of early mortality (HRs, 95% CIs: 0.85, [0.74,0.98]; 0.86, [0.74,0.98], 0.86, [0.75,0.99], 0.86, [0.75,0.99]).

The four specifications of Table 3 consider NFL age-at-entry as a categorical variable but are otherwise identical to those of Table 2.

The results of the Table 3 regressions are consistent with those of Table 2. Namely, not being in the first quartile of NFL age-at-entry has a significant protective effect upon a player’s mortality hazard (HRs, 95% CIs: 0.75, [0.58,0.97]; 0.75, [0.58,0.97]; 0.76, [0.59,0.98]; 0.76, [0.59,0.98]).

## 4. Discussion

These results suggest that an advanced NFL age-at-entry is protective against early mortality, even when conditioning upon player Draft position, career game exposure, position-of-play group, BMI, and birth year or decade. Specifically, a one-year increase in age at NFL entry is associated with a 14% decreased hazard of death. This protective effect is found both when variation in age-at-entry is represented continuously and when it is represented categorically. While this study is novel in its consideration of age-at-entry into the NFL, it is consistent with findings that an earlier age-at-entry into youth football is associated with increased risk of long-term health risks [1,2,3,4,5,6]. Indeed, younger NFL players face greater cumulative head impact risk than at any other level of play (e.g., than their age peers who remain in college football) and a lower level of expected cognitive development than their older League peers in the NFL.

Methodologically, the specification of position cluster indicator variables and games played has certain advantages over the CHII approach to controlling for position-related early mortality risk. Whereas CHII depends upon helmet accelerometer studies to adjust for risk and thus does not include special teams players, the present approach has no such limitation and thus can be specified for all player positions. Moreover, position cluster indicator variables do not depend upon the correctness of a fitted formula, unlike CHII. Rather, they represent a black-box catch-all for position cluster-specific factors that influence earlier mortality risk; player-position indicator variables measure variation in this risk based on the observed data, without any need for functional specification. On the other hand, CHII estimates depend upon the specification/assumption of a specific functional form that multiplies games played by a positional risk adjustment. The results suggest a new path toward a refined CHII measure, one that incorporates position indicator variable coefficient estimates. In addition to position controls, which have been shown to relate to head impact exposure, we also control for BMI and era of play to control out noise when estimating the relationship between age-at-entry and mortality.

Expected ability upon League entry, as defined by draft round selected, is an important control, as it may indicate number of expected snaps per game played, [25,26] which was not tracked well until recent seasons. For example, some backup players might play in games regularly during their career but may have a relatively low snap count per game (e.g., if they are used in limited, special teams roles). Prior studies use the number of games and seasons played as a proxy for a player’s exposure to play. However, such variables alone omit variation in risk exposure per game played. This omission is difficult to rectify, as even the number of games started has been tracked only in recent decades. There is strong reason to believe that Draft round of player selection, conditional upon position of play, indicates greater expected snap exposure per game [20,21].

Given the cohort design of the study, the analysis represents, essentially, a census analysis of players entering the NFL between 1970 and 2017. As such, our study avoids the sample selection-bias issues of other sampling methods that have been used to study the post-career health of NFL players (e.g., for those that rely upon data of pension-eligible players only, where pension eligibility depends upon years played).

## 5. Conclusions

Most directly, the results present strong evidence in favor of the implementation of a minimum eligibility age for NFL entry. Our results suggests that an age eligibility floor, imposed at 22 to 23 years old, would likely prevent significant earlier mortality among NFL players. The results also suggest that the NFL’s present indirect age-at-entry policy—that a player is eligible to play in the NFL three years subsequent to high school graduation—is inadequate from the standpoint of player protection. This policy has allowed players to be drafted into the League before turning 20 years old and players as young as 20.2 years to play in an NFL game. As such, many younger players enter the League prior to neurobiological adulthood and, therefore, face greater short and long-term neurophysiological effects from head impact [12,13,14]. The central finding of the present study—an earlier all-cause mortality effect of early NFL entry—is consistent with these previous findings. These respective results may be related, although further research involving cause-specific mortality is needed to assess the pathway from neurophysiological effects to mortality effects.

Historically, there has been pressure to remove or relax the present NFL player eligibility rule of at least three years post-high school graduation. College players have challenged the rule as restricting talented younger players from NFL labor market access in potential violation of the Sherman Anti-Trust Act. In Clarett v. NFL 2004, a U.S. District Court ruled that the NFL’s standing policy violates the Sherman Anti-Trust Act. However, the ruling was later overturned by current U.S. Supreme Court Justice Sonia Sotomayor, who was then a U.S. Court of Appeals Justice. In a Forbes article, Edelman writes that the case “marked a watershed moment in sports law history as, for a brief moment, it called into doubt the NFL’s age/education requirement” [27]. The results of the present study suggest that a relaxation of the present NFL player eligibility rule would lead to further influx of players who are under 22 years old and a resulting aggregate escalation in the observed early mortality risk presented by NFL exposure for these younger players. A growing body of research suggests that interventions by teams and leagues can reduce the cumulative head impact exposure to young players [28,29,30].

This research finds evidence that early mortality risk factors presented by exposure-to-play (e.g., head impact exposure) may not be uniform across one’s career. Future research can address the extent to which the age at which head impact is incurred influences the associated mortality risk. Limits of the study include lack of information on cause-specific mortality in the data. Such determination would allow us to study age-at-entry for specific causes of death.

The practical implications of the study are as follows: age-at-entry represents a choice variable for the NFL. For example, the NBA enforces an explicit age for league entry. This study finds significant evidence that an advanced age at league entry is protective of mortality in a study that features comprehensive controls. In a high-impact sport, such as American football, it is vital to continue examining the age-specific impacts of play and the age-specific impacts of level-of-play upon player health. Such a continued examination will allow for the assessment of player risks at each phase of one’s playing career.

## Figures and Tables

**Table 1:** National Football League Player Demographics (Players Entering 1970–2017; *n* = 9049).

Variable	Total (*n* = 9049)	QBs (*n* = 433)	WRs (*n* = 1806)	Offensive Backs or RBs (*n* = 1126)	Defensive Backs (*n* = 1732)	LBs (*n* = 1323)	Offensive Linemen (*n* = 1034)	Defensive Linemen (*n* = 1408)	Special Teams (*n* = 187)
Age, mean (SD), y at NFL entry	22.9 (0.8)	23.0 (0.8)	22.8 (0.8)	22.8 (0.9)	22.8 (0.8)	22.8 (0.8)	22.9 (0.8)	22.9 (0.9)	23.0 (1.0)
Seasons in NFL, median (IQR)	5 (3–8)	5 (2–9)	4 (2–7)	4 (2–6)	5 (3–8)	5 (3–8)	7 (4–9)	5 (3–8)	3 (2–7)
Age, mean (SD), y as of 7/1/2018	46.6 (13.5)	48.0 (13.3)	46.8 (13.5)	49.2 (13.5)	44.8 (13.3)	47.0 (13.4)	48.0 (13.4)	44.2 (13.3)	51.3 (13.5)
Dead, *n* (%)	365 (4.0%)	12 (2.8%)	56 (3.1%)	64 (5.7%)	51 (2.9%)	49 (3.7%)	43 (4.2%)	84 (6.0%)	6 (3.2%)
Age, mean (SD), y at death	47.4 (12.4)	51.2 (10.0)	48.6 (12.5)	48.9 (12.0)	46.2 (13.8)	43.4 (12.2)	50.7 (12.1)	45.9 (11.9)	54.3 (8.4)
BMI, mean (SD), kg/m^2^	29.8 (4.0)	26.9 (1.5)	27.3 (2.5)	29.4 (2.2)	26.5 (1.5)	30.3 (1.7)	35.2 (3.1)	34.3 (3.4)	26.4 (1.8)
Height, mean (SD), cm	187.1 (6.4)	190.1 (4.0)	186.6 (6.7)	181.5 (4.6)	182.1 (4.1)	188.0 (3.6)	193.9 (4.0)	192.2 (4.1)	183.7 (5.6)

*n* = 9049; Percent dead compared using a χ^2^ test; age, age at death, body mass index (BMI), and height compared using ANOVA. All characteristics except age-at-death were statistically significantly different across the positions (*p* < 0.001), where age-at-death was statistically significant at α=0.05.

**Table 2:** Cox Regression Results with NFL Age-at-Entry Specified.

Variables	(1)	(2)	(3)	(4)
Age-at-entry	0.85	0.86	0.86	0.86
C.I.	(0.74–0.98)	(0.74–0.98)	(0.75–0.99)	(0.75–0.99)
*p*-value	0.027	0.027	0.038	0.037
Birth Year	0.99	0.99		
C.I.	(0.98–1.00)	(0.98–1.00)		
*p*-value	0.177	0.190		
born40s			2.92	2.96
C.I.			(0.38–22.28)	(0.39–22.53)
*p*-value			0.300	0.296
born50s			2.81	2.82
C.I.			(0.37–21.08)	(0.38–21.21)
*p*-value			0.316	0.313
born60s			2.24	2.30
C.I.			(0.30–16.80)	(0.31–17.28)
*p*-value			0.432	0.417
born70s			1.61	1.65
C.I.			(0.21–12.20)	(0.22–12.48)
*p*-value			0.643	0.628
born80s			2.88	2.91
C.I.			(0.38–21.78)	(0.38–21.95)
*p*-value			0.305	0.301
BMI	1.11	1.11	1.12	1.12
C.I.	(1.07–1.16)	(1.07–1.16)	(1.08–1.16)	(1.08–1.16)
*p*-value	0.000	0.000	0.000	0.000
Quarterback	0.93	0.94	0.95	0.95
C.I.	(0.35–2.50)	(0.35–2.51)	(0.35–2.54)	(0.35–2.55)
*p*-value	0.890	0.897	0.916	0.920
Wide Receiver	0.70	0.66	0.69	0.64
C.I.	(0.29–1.72)	(0.27–1.61)	(0.28–1.68)	(0.26–1.58)
*p*-value	0.439	0.358	0.408	0.336
Running Back	1.36	1.29	1.36	1.30
C.I.	(0.58–3.18)	(0.55–3.02)	(0.59–3.18)	(0.56–3.03)
*p*-value	0.473	0.552	0.472	0.547
Defensive Back	1.09	1.05	1.10	1.06
C.I.	(0.47–2.54)	(0.45–2.44)	(0.47–2.57)	(0.46–2.48)
*p*-value	0.839	0.912	0.818	0.888
Linebacker	1.37	1.27	1.34	1.25
C.I.	(0.54–3.50)	(0.50–3.25)	(0.53–3.44)	(0.49–3.21)
*p*-value	0.505	0.611	0.537	0.638
Offensive Lineman	0.99	0.93	0.99	0.94
C.I.	(0.42–2.34)	(0.40–2.21)	(0.42–2.34)	(0.40–2.21)
*p*-value	0.987	0.877	0.986	0.883
Defensive Lineman	1.38	1.30	1.38	1.31
C.I.	(0.60–3.16)	(0.56–2.98)	(0.60–3.18)	(0.57–3.01)
*p*-value	0.453	0.541	0.445	0.528
Games	1.00	1.00	1.00	1.00
C.I.	(1.00–1.00)	(1.00–1.01)	(1.00–1.00)	(1.00–1.01)
*p*-value	0.034	0.213	0.040	0.248
Games squared		1.00		1.00
C.I.		(1.00–1.00)		(1.00–1.00)
*p*-value		0.048		0.062
Draft Round 1	1.37	1.34	1.37	1.34
C.I.	(0.98–1.90)	(0.96–1.86)	(0.98–1.90)	(0.96–1.86)
*p*-value	0.064	0.083	0.064	0.081
Draft Round 2	1.19	1.16	1.20	1.16
C.I.	(0.85–1.68)	(0.82–1.63)	(0.85–1.68)	(0.83–1.64)
*p*-value	0.312	0.406	0.299	0.383
Draft Round 3	0.95	0.93	0.95	0.94
C.I.	(0.65–1.37)	(0.64–1.35)	(0.66–1.38)	(0.65–1.36)
*p*-value	0.768	0.691	0.802	0.734
Draft Round 4	1.25	1.22	1.26	1.23
C.I.	(0.87–1.78)	(0.85–1.74)	(0.88–1.79)	(0.86–1.76)
*p*-value	0.223	0.274	0.210	0.254
Draft Round 5	1.10	1.08	1.11	1.09
C.I.	(0.75–1.61)	(0.73–1.58)	(0.75–1.63)	(0.74–1.60)
*p*-value	0.641	0.710	0.602	0.664
Draft Round 6	0.99	0.98	1.00	0.99
C.I.	(0.65–1.52)	(0.64–1.50)	(0.65–1.53)	(0.65–1.52)
*p*-value	0.965	0.926	0.996	0.963
Observations	9049	9049	9049	9049

Positions 1 = quarterback; Positions 2 = wide receiver; Positions 3 = offensive back or running back; Positions 4 = defensive back; Positions 5 = linebacker; Positions 6 = offensive lineman; Positions 7 = defensive lineman; Positions 8 = reference group = special teams players.

**Table 3:** Cox Regression Results with Not NFL Entry Age Q1 Specified.

Variables	(5)	(6)	(7)	(8)
Not Entry Age Q1	0.75	0.75	0.76	0.76
C.I.	(0.58–0.97)	(0.58–0.97)	(0.59–0.98)	(0.59–0.98)
*p*-value	0.028	0.028	0.034	0.034
Birth Year	0.99	0.99		
C.I.	(0.98–1.00)	(0.98–1.00)		
*p*-value	0.176	0.190		
born40s			2.85	2.88
C.I.			(0.37–21.73)	(0.38–21.93)
*p*-value			0.312	0.307
born50s			2.80	2.81
C.I.			(0.37–21.04)	(0.37–21.14)
*p*-value			0.317	0.314
born60s			2.22	2.28
C.I.			(0.30–16.63)	(0.30–17.08)
*p*-value			0.438	0.423
born70s			1.58	1.61
C.I.			(0.21–11.91)	(0.21–12.18)
*p*-value			0.660	0.644
born80s			2.85	2.86
C.I.			(0.38–21.49)	(0.38–21.63)
*p*-value			0.311	0.308
BMI	1.11	1.11	1.12	1.12
C.I.	(1.07–1.15)	(1.07–1.15)	(1.07–1.16)	(1.08–1.16)
*p*-value	0.000	0.000	0.000	0.000
Quarterback	0.93	0.93	0.94	0.94
C.I.	(0.35–2.49)	(0.35–2.49)	(0.35–2.53)	(0.35–2.53)
*p*-value	0.885	0.886	0.907	0.906
Wide Receiver	0.71	0.67	0.69	0.65
C.I.	(0.29–1.75)	(0.27–1.63)	(0.28–1.69)	(0.26–1.59)
*p*-value	0.461	0.374	0.420	0.344
Running Back	1.37	1.30	1.37	1.30
C.I.	(0.59–3.20)	(0.56–3.02)	(0.59–3.19)	(0.56–3.03)
*p*-value	0.467	0.549	0.470	0.548
Defensive Back	1.09	1.04	1.10	1.06
C.I.	(0.47–2.54)	(0.45–2.43)	(0.47–2.56)	(0.45–2.46)
*p*-value	0.843	0.921	0.825	0.900
Linebacker	1.39	1.28	1.35	1.25
C.I.	(0.54–3.54)	(0.50–3.28)	(0.53–3.45)	(0.49–3.21)
*p*-value	0.491	0.601	0.536	0.641
Offensive Lineman	1.01	0.94	1.00	0.94
C.I.	(0.43–2.37)	(0.40–2.23)	(0.42–2.36)	(0.40–2.23)
*p*-value	0.990	0.894	0.999	0.893
Defensive Lineman	1.37	1.29	1.38	1.30
C.I.	(0.60–3.16)	(0.56–2.98)	(0.60–3.17)	(0.56–3.00)
*p*-value	0.455	0.547	0.450	0.536
Games	1.00	1.00	1.00	1.00
C.I	(1.00–1.00)	(1.00–1.01)	(1.00–1.00)	(1.00–1.01)
*p*-value	0.046	0.198	0.052	0.236
Games squared		1.00		1.00
C.I.		(1.00–1.00)		(1.00–1.00)
*p*-value		0.048		0.063
Draft Round 1	1.39	1.36	1.39	1.36
C.I.	(1.00–1.93)	(0.98–1.89)	(1.00–1.93)	(0.98–1.89)
*p*-value	0.051	0.068	0.052	0.067
Draft Round 2	1.20	1.17	1.21	1.18
C.I.	(0.86–1.69)	(0.83–1.64)	(0.86–1.70)	(0.84–1.65)
*p*-value	0.287	0.376	0.273	0.353
Draft Round 3	0.95	0.93	0.96	0.94
C.I.	(0.65–1.38)	(0.64–1.35)	(0.66–1.39)	(0.65–1.37)
*p*-value	0.788	0.708	0.824	0.753
Draft Round 4	1.25	1.22	1.26	1.24
C.I.	(0.88–1.79)	(0.86–1.75)	(0.88–1.80)	(0.86–1.77)
*p*-value	0.216	0.267	0.203	0.246
Draft Round 5	1.11	1.09	1.12	1.10
C.I.	(0.75–1.63)	(0.74–1.60)	(0.76–1.65)	(0.75–1.62)
*p*-value	0.595	0.666	0.559	0.622
Draft Round 6	0.99	0.98	1.00	0.99
C.I.	(0.65–1.52)	(0.64–1.50)	(0.65–1.53)	(0.65–1.52)
*p*-value	0.964	0.924	0.998	0.964
Observations	9049	9049	9049	9049

Positions 1 = quarterback; Positions 2 = wide receiver; Positions 3 = offensive back or running back; Positions 4 = defensive back; Positions 5 = linebacker; Positions 6 = offensive lineman; Positions 7 = defensive lineman; Positions 8 = reference group = special teams players.

## Data Availability

The data that support the findings of this study are available from a cited online source.

## References

[1] Alosco M.L., Kasimis A.B., Stamm J.M., Chua A., Baugh C.M., Daneshvar D., Robbins C., Mariani M., Hayden J., Conneely S. (2017). Age of first exposure to American football and long-term neuropsychiatric and cognitive outcomes. Transl. Psychiatry.

[2] Alosco M.L., Mez J., Tripodis Y., Kiernan P.T., Abdolmohammadi B., Murphy L., Kowall N.W., Stein T.D., Huber B.R., Goldstein L.E. (2018). Age of first exposure to tackle football and chronic traumatic encephalopathy. Ann. Neurol..

[3] AMA (2020). Traumatic Brain Injuries Can Lead to Long-Term Neurological and Psychiatric Disorders. ScienceDaily. https://www.sciencedaily.com/releases/2018/11/181102083501.htm.

[4] Stamm J.M., Bourlas A.P., Baugh C.M., Fritts N.G., Daneshvar D.H., Martin B.M., McClean M.D., Tripodis Y., Stern R.A. (2015). Age of first exposure to football and later-life cognitive impairment in former NFL players. Neurology.

[5] Tagge C.A., Fisher A.M., Minaeva O.V., Gaudreau-Balderrama A., Moncaster J., Zhang X.-L., Wojnarowicz M.W., Casey N., Lu H., Kokiko-Cochran O.N. (2018). Concussion, microvascular injury, and early tauopathy in young athletes after impact head injury and an impact concussion mouse model. Brain.

[6] Talavage T.M., Nauman E.A., Breedlove E.L., Yoruk U., Dye A.E., Morigaki K.E., Feuer H., Leverenz L.J. (2014). Functionally-Detected Cognitive Impairment in High School Football Players without Clinically-Diagnosed Concussion. J. Neurotrauma.

[7] Alosco M.L., Stern R.A. (2019). Youth Exposure to Repetitive Head Impacts from Tackle Football and Long-term Neurologic Outcomes: A Review of the Literature, Knowledge Gaps and Future Directions, and Societal and Clinical Implications. Semin. Pediatr. Neurol..

[8] Deshpande S.K., Hasegawa R.B., Rabinowitz A.R., Whyte J., Roan C.L., Tabatabaei A., Baiocchi M., Karlawish J.H., Master C., Small D.S. (2017). Association of Playing High School Football with Cognition and Mental Health Later in Life. JAMA Neurol..

[9] Yang J., Comstock R.D., Yi H., Harvey H.H., Xun P. (2017). New and recurrent concussions in high-school athletes before and after traumatic brain injury laws, 2005–2016. Am. J. Public Health.

[10] Baillargeon A., Lassonde M., Leclerc S., Ellemberg D. (2012). Neuropsychological and neurophysiological assessment of sport concussion in children, adolescents and adults. Brain Inj..

[11] Silveri M.M. (2012). Adolescent Brain Development and Underage Drinking in the United States: Identifying Risks of Alcohol Use in College Populations. Harv. Rev. Psychiatry.

[12] Pellman E.J., Lovell M.R., Viano D.C., Casson I.R. (2006). Concussion in Professional Football: Recovery of NFL and High School Ath-letes Assessed by Computerized Neuropsychological Testing—Part 12. Neurosurgery.

[13] Daniel R.W., Rowson S., Duma S.M. (2012). Head Impact Exposure in Youth Football. Ann. Biomed. Eng..

[14] Schnebel B., Gwin J.T., Anderson S., Gatlin R. (2007). In vivo study of head impacts in football: A comparison of National Collegiate Athletic Association Division I versus High School Impacts. Neurosurgery.

[15] Nathanson J.T., Connolly J.G., Yuk F., Gometz A., Rasouli J., Lovell M., Choudhri T. (2016). Concussion incidence in professional football: Position-specific analysis with use of a novel metric. Orthop. J. Sports Med..

[16] NCAA What Sport Has the Most Concussions?|Concussion Rate. Complete Concussion Management Inc. Published 6 December 2018. https://completeconcussions.com/2018/12/05/concussion-rates-what-sport-most-concussions/.

[17] Gerberich S.G., Priest J.D., Boen J.R., Straub C.P., Maxwell R.E. (1983). Concussion incidences and severity in secondary school varsity football players. Am. J. Public Health.

[18] Harrison E.A. (2014). The First Concussion Crisis: Head Injury and Evidence in Early American Football. Am. J. Public Health.

[19] Harvey H.H. (2013). Reducing Traumatic Brain Injuries in Youth Sports: Youth Sports Traumatic Brain Injury State Laws, January 2009–December 2012. Am. J. Public Health.

[20] Baugh C.M., Kroshus E. (2016). Concussion management in US college football: Progress and pitfalls. Concussion.

[21] Kerr Z.Y., Register-Mihalik J.K., Pryor R., Pierpoint L.A., Scarneo S.E., Adams W.M., Kucera K.L., Casa D.J., Marshall S.W. (2019). The Association between Mandated Preseason Heat Acclimatization Guidelines and Exertional Heat Illness during Preseason High School American Football Practices. Environ. Health Perspect..

[22] Montenigro P., Alosco M.L., Martin B.M., Daneshvar D., Mez J., Chaisson C.E., Nowinski C.J., Au R., McKee A.C., Cantu R.C. (2017). Cumulative Head Impact Exposure Predicts Later-Life Depression, Apathy, Executive Dysfunction, and Cognitive Impairment in Former High School and College Football Players. J. Neurotrauma.

[23] Kmush B.L., Mackowski M., Ehrlich J., Walia B., Owora A., Sanders S. (2020). Association of Professional Football Cumulative Head Impact Index Scores with All-Cause Mortality Among National Football League Players. JAMA Netw. Open.

[24] StataCorp Stata statistical software: Release 14. StataCorp LP: 2015. https://www.stata.com/stata14/.

[25] Camerer C.F., Weber R.A. (1999). The econometrics and behavioral economics of escalation of commitment: A re-examination of Staw and Hoang’s NBA data. J. Econ. Behav. Organ..

[26] Staw B.M., Hoang H. (1995). Sunk Costs in the NBA: Why Draft Order Affects Playing Time and Survival in Professional Basketball. Adm. Sci. Q..

[27] Edelman M. The NFL Age Requirement Was Briefly “Sacked” Ten Years Ago Today. https://www.forbes.com/sites/marcedelman/2014/02/05/the-nfl-age-requirement-was-briefly-sacked-ten-years-ago-today/#761cc03b64a.

[28] Broglio S.P., Martini D., Kasper L., Eckner J.T., Kutcher J.S. (2013). Estimation of head impact exposure in high school football: Implications for regulating contact practices. Am. J. Sports Med..

[29] Clark M., Asken B., Marshall S.W., Guskiewicz K.M. (2017). Descriptive Characteristics of Concussions in National Football League Games, 2010–2011 to 2013–2014. Am. J. Sports Med..

[30] Venkataramani A.S., Gandhavadi M., Jena A.B. (2018). Association Between Playing American Football in the National Football League and Long-term Mortality. JAMA.

